# Partial Splenic Embolization as a Bridge to Total Knee Replacement for a Patient with Severe Thrombocytopenia due to Cirrhosis and Splenic Sequestration

**DOI:** 10.1155/2012/317807

**Published:** 2012-12-04

**Authors:** Adrianne Netterville, Ronald Lands

**Affiliations:** ^1^Department of Medicine, University of Tennessee, 1924 Alcoa Hwy U-114, Knoxville, TN 37924, USA; ^2^Division of Hematology, Department of Medicine, University of Tennessee, 1924 Alcoa Hwy U-114, Knoxville, TN 37894, USA

## Abstract

Medical splenectomy by embolization was originally used to attenuate bleeding from varices in a man with cirrhosis and portal hypertension. Despite the procedure being described over 40 years ago with remarkable improvement in its safety profile and clinical outcomes since, it is still used with variable frequency because of concerns that the risk is high and the results are transient. We present the case of an elderly woman with cirrhosis, portal hypertension, and splenic sequestration who completed partial splenic embolization (PSE) with a durable hematologic response that served as a bridge which allowed her to have orthopedic surgery. A discussion with literature review follows.

## 1. Introduction

The management of hematologic abnormalities in the surgical patient who suffers from chronic liver disease is challenging. The need for adequate intraoperative clotting must be balanced with the almost counterintuitive need to consider postoperative deep vein thrombosis and pulmonary embolism (DVT/PE) prophylaxis. We present a patient with this clinical dilemma who asked for a total knee replacement (TKR) in hopes that it would improve her mobility, and thus, her quality of life. PSE mitigated her thrombocytopenia and allowed her to have a TKR with adequate hemostasis and standard postoperative DVT/PE prophylaxis.

## 2. Case Presentation

A 76-year-old woman with untreated hepatitis C, cirrhosis, portal hypertension, pancytopenia, and severe thrombocytopenia asked for a total knee replacement. She had survived a near lethal car accident in the 1980s and required multiple transfusions of blood products. She contracted hepatitis C and after not responding to interferon, had not been treated again. A few years after the accident, she required a left below the knee amputation because of chronic osteomyelitis. She had no history of excessive bruising or bleeding from any orifice. Physical exam demonstrated telangiectasias scattered over her upper chest wall. Neither the liver nor the spleen was palpable. The right knee was fixed in a flexed position at nearly a 90 degree angle rendering her wheelchair bound.

Her initial CBC demonstrated a hemoglobin of 9.9 g/dL with total WBC of 1.9 × 10^3^ and platelet count of 34 × 10^3^. Review of the smear did not demonstrate schistocytes, acanthocytes, or target cells. There were 1-2 platelets per oil immersion field. The creatinine was 0.6 mg/dL, total bilirubin 0.7 mg/dL, and albumin 2.8 g/dL. The PT was 12.7 seconds, PTT 31.1 seconds, fibrinogen 192 mg/dL, and the thrombin time 19 seconds. Antithrombin III function was 54%, Factor V 66%, Factor VII 79%, Factor VIII 120%. Rheumatoid factor, ANA, and cryoglobulins were not detected. The bone marrow histology demonstrated a cellular marrow without infiltration by malignant cells or evidence of myelodysplasia. The spleen was 15 cm by ultrasound, an increase of 5 cm when compared to a previous scan taken three years earlier.

After much discussion regarding the potential risks and desired benefits, she agreed to PSE. An interventional radiologist at our medical center with significant experience performing PSE embolized 1/2–2/3 of the total volume of her spleen. Five days after the procedure, she had to be admitted to the hospital for fever, shortness of breath, left lower lobe infiltrate, and effusion. While she was treated for a community acquired pneumonia, it is more likely that she had a postembolization syndrome. Her platelet count was 129 × 10^3^ and her WBC was 14 × 10^3^ when she was discharged home to finish recuperating after 4 days. One month after her PSE, her platelet count was 140 × 10^3^. She was referred back to her surgeon who subsequently performed the TKR without bleeding complications and with standard postoperative deep vein thrombosis prophylaxis. Her blood parameters are stable one year after the procedure. 

## 3. Discussion

Surgery and anesthesia in patients with chronic liver disease is fraught with many potential complications [1]. Metabolic insufficiency hampers the ability of the liver to manage toxins or metabolize drugs. Inadequate synthesis of clotting factors increases the risk of bleeding. The impaired liver dependent synthesis of the anticoagulant proteins C and S under ordinary conditions creates a hypercoagulable state. The confluence of these opposing abnormalities may produce a situation where the PT/INR may not accurately predict the risk of either bleeding or clotting. Administration of vitamin K and plasma transfusions attenuate the bleeding risk, but the results are transient and often incomplete.

Factors unrelated to the synthetic or metabolic functions of the liver may add to the hematologic abnormalities. Thrombocytopenia may be caused by one or a combination of hematologic aberrations. It is often difficult to separate worsening liver function from the development of disseminated intravascular coagulation (DIC). Especially In the elderly, bone marrow failure syndromes such as myelodysplasia may confuse the picture. Untreated hepatitis C introduces several variables including bone marrow suppression and autoimmune destruction of the platelets [2, 3]. Portal hypertension and splenic sequestration often contributes to pancytopenia.

Maddison first reported the use of splenic embolization in a man with bleeding esophageal varices who demonstrated improvement in his hematologic parameters and cessation of his hemorrhage [4]. Attempts to duplicate this success resulted in serious complications including sepsis, splenic abscess, splenic rupture, or death. Complications were later shown to be related to the amount of spleen embolized with volumes more than 60–70% being associated with an increased risk of morbidity or death [5–7]. Outcomes improved with the use of partial rather than total splenic embolization, strict antibiotic technique, antibiotic prophylaxis, pain control, and careful postembolization care [8].

While the improvement in hematologic parameters is often attributed to changes in the spleen pool with reduced spleen size, there may be an immunologic mechanism. Reductions in platelet associated immunoglobulin G were also associated with reduction in spleen size and improved platelet counts. There is not a good explanation for the improvement in leukocyte counts or the lack of response in the hemoglobin [9]. 

In addition to improvement in the hematologic parameters, some studies indicate that liver function also improves. A recent review of the literature seemed to indicate that PSE reduces bleeding, improves hematologic parameters as well as protein synthesis, and decreases the severity of hepatic encephalopathy [10]. Improvement in total protein, total cholesterol, and prothrombin time improved significantly in patients with Childs A and B, but not in Child's class C liver disease, and was durable for at least one year [11]. Another study demonstrated a median survival of 50 months with improvement in hematologic parameters, reduction in bleeding episodes from esophageal varices, and improved clinical status contributing to symptom control [12]. Improved hepatic blood flow may account for the improvement in liver function [9].

While the original focus of total and PSE was to attenuate the effects of bleeding and encephalopathy, the hematologic improvements provide opportunities in a variety of other clinical scenarios. Oncologists have exploited the increased leukocyte and platelet counts after PSE to administer chemotherapy without dose reductions to patients with liver disease and cancer [13, 14]. PSE has been used to mitigate the bone marrow suppression of ribavirin in patients treated for Hepatitis C [15]. It successfully improved coagulation parameters in a patient needing neurosurgery [16]. 

Our patient, while elderly and suffering with chronic liver disease, successfully completed PSE to attenuate her thrombocytopenia and safely complete TKR without excessive bleeding and with standard postoperative DVT/PE prophylaxis. PSE can be used as bridge therapy to orthopedic surgery for elderly patients with thrombocytopenia due to sequestration.

## References

[1] Hanje AJ, Patel T (2007). Preoperative evaluation of patients with liver disease. *Nature Clinical Practice Gastroenterology and Hepatology*.

[2] Olariu M, Olariu C, Olteanu D (2010). Thrombocytopenia in chronic hepatitis C. *Journal of Gastrointestinal and Liver Diseases*.

[3] Wang CS, Yao WJ, Wang ST, Chang TT, Chou P (2004). Strong association of hepatitis C virus (HCV) infection and thrombocytopenia: implications from a survey of a community with hyperendemic HCV infection. *Clinical Infectious Diseases*.

[4] Maddison F (1973). Embolic therapy of hypersplenism. *Investigative Radiology*.

[5] Mozes MF, Spigos DG, Pollak R (1984). Partial splenic embolization, an alternative to splenectomy—results of a prospective, randomized study. *Surgery*.

[6] N'Kontchou G, Seror O, Bourcier V (2005). Partial splenic embolization in patients with cirrhosis: efficacy, tolerance and long-term outcome in 32 patients. *European Journal of Gastroenterology and Hepatology*.

[7] Sakai T, Shiraki K, Inoue H (2002). Complications of partial splenic embolization in cirrhotic patients. *Digestive Diseases and Sciences*.

[8] Spigos DG, Jonasson O, Mozes M, Capek V (1979). Partial splenic embolization in the treatment of hypersplenism. *American Journal of Roentgenology*.

[9] Sakata K, Hirai K, Tanikawa K (1996). A long-term investigation of transcatheter splenic arterial embolization for hypersplenism. *Hepato-Gastroenterology*.

[10] Koconis KG, Singh H, Soares G (2007). Partial splenic embolization in the treatment of patients with portal hypertension: a review of the english language literature. *Journal of Vascular and Interventional Radiology*.

[11] Murata K, Shiraki K, Takase K, Nakano T, Tameda Y (1996). Long term follow-up for patients with liver cirrhosis after partial splenic embolization. *Hepato-Gastroenterology*.

[12] Pålsson B, Hallén M, Forsberg AM, Alwmark A (2003). Partial splenic embolization: long-term outcome. *Langenbeck’s Archives of Surgery*.

[13] Lokich J, Costello P (1983). Splenic embolization to prevent dose limitation of cancer chemotherapy. *American Journal of Roentgenology*.

[14] Kauffman CR, Mahvash A, Kopetz S, Wolff RA, Ensor J, Wallace MJ (2008). Partial splenic embolization for cancer patients with thrombocytopenia requiring systemic chemotherapy. *Cancer*.

[15] Foruny JR, Blázquez J, Moreno A (2005). Safe use of pegylated interferon/ribavirin in hepatitis C virus cirrhotic patients with hypersplenism after partial splenic embolization. *European Journal of Gastroenterology and Hepatology*.

[16] Pinto AG, Namyslowski J, Pandya P (2005). Severe thrombocytopenia due to hypersplenism successfully treated with partial splenic embolization in preoperative management. *Southern Medical Journal*.

